# Peripheral adenomatoid odontogenic tumor in a cloak of an epulis: report of a rare case

**DOI:** 10.1186/s12903-019-0759-8

**Published:** 2019-05-10

**Authors:** Dhara Dwivedi, Nitin Prabhakar, Sowmya Kasetty, Rinky Ahuja

**Affiliations:** 1 Department of Oral Pathology, Dentistry Unit, Ayder Referral Hospital- College of Health Sciences, CHS Mekelle University, Mekelle, Ethiopia; 2 Department of Oral Maxillofacial Surgery, Ayder referral hospital- College of Health Sciences, Mekelle university, Mekelle, Ethiopia; 3 Oral Pathology Division, Oral Basic and Clinical Sciences, Qassim Private College, Buraidah, Kingdom of Saudi Arabia; 4 People’s College of Dental Sciences & Research Centre, Bhopal, Madhya Pradesh, Bhopal, India

**Keywords:** Adenomatoid odontogenic tumor, Peripheral odontogenic tumors, Peripheral adenomatoid odontogenic tumor, Gingival lesions

## Abstract

**Background:**

Adenomatoid odontogenic tumor constitutes an uncommon benign odontogenic tumor which is well-known as “two-thirds tumor” (two-thirds of adenomatoid tumors occur in the maxilla, two-thirds occur in young females, two-thirds of the cases are associated with un-erupted teeth and two-thirds of the affected teeth are canines). Larger part of these present as intra - osseous tumors while peripheral counterparts are extremely rare. Peripheral adenomatoid odontogenic tumor is a unique entity which generally presents as a slow growing gingival swelling with minimal bone involvement. This often leads to its erroneous diagnosis as a simple gingival lesion and the real diagnosis is only revealed after its microscopic evaluation. It exhibits a marked predilection for maxillary gingiva of incisor region and most commonly affects the younger females.

**Case presentation:**

We report a case of 25 years old female patient of African ethnicity who presented with a 2 × 2 cm mass attached to the left mandibular gingiva in cuspid- bicuspid region which is an unusual site for AOT. It was accompanied by slight bone loss. With the differential diagnosis of gingival epulis and peripheral ossifying fibroma; surgical excision was performed. The light microscopic examination of the specimen aided the final diagnosis of Adenomatoid odontogenic tumor with the histopathological features identical to its intra osseous counterpart.

**Conclusion:**

In this case, the tumor is present on the mandibular gingiva in a 25 years old patient which is an atypical location and age for this tumor; also, the only individual case reported in an African patient. Only, a meager number of peripheral adenomatoid odontogenic tumor cases have been logged so far with majority of them occurring in maxillary gingiva. Furthermore, an ambiguity still prevails regarding its true origin and possible biological course. Hence, reporting of similar cases should be encouraged to facilitate the better understanding of its varied epidemiological details and clinical presentation.

## Background

The term Adenomatoid odontogenic tumor (AOT) was given by Philipsen and Birn in 1969. It has described by numerous authors under diverse terminology like “adamantoma”, “epithelial odontome”, “cystic adamantoma”, “adenoameloblastoma”, “tooth germ (or chorioblastomatous) cyst of the jaw”, “epithelial tumors associated with developmental cysts of the maxilla” and several more dating from 1877 [1–4]. This makes it difficult to determine the specific year of when the case of AOT was reported for the first time. The term AOT was accepted in the initial edition of the World Health Organization’s (WHO) Histological Typing of Odontogenic Tumors, Jaw Cysts and Allied Lesions in 1971 and has been retained since then [5–9].

AOT is a relatively rare distinct odontogenic neoplasm accounting for 2.2–7.1% of all odontogenic tumours. It constitutes about 1.2% of all odontogenic tumors (OTs) in Caucasians and up to 9% of OTs in Black Africans. The three clinicopathological variants of AOT are: follicular type, extrafollicular type and peripheral variety. Follicular and extrafollicular variants account for 97.7% of all the AOTs and are intrabony tumors while peripheral type is the rarest of them all constituting only 2.3% of all AOTs [10–12]. The follicular variant is invariably associated with an unerupted tooth and presents as a well-defined, unilocular radiolucency associated with the crown and often part of the root of an unerupted tooth; thus, mimicking the dentigerous cyst. On the other hand, extra follicular variant has no association with unerupted tooth and presents as a well-defined, unilocular radiolucency found between, above or superimposed upon the roots of erupted permanent teeth. Peripheral adenomatoid odontogenic tumor (POAT) is the tumor that demonstrates the histologic characteristics of its intraosseous counterparts but occurs solely in the soft tissue covering the tooth-bearing portion of the mandible and maxilla [8, 13].

Similar to the central AOT, the subject of histiogenesis of PAOT also remains unsettled. Two theories have been proposed in regard to the origin of PAOT presenting as gingival mass. On one hand, PAOT with no to minimal bone loss is suggestive of its de novo origin; on the other hand, notable bone involvement can be indicative of its origin as tumor arising intraosseously that is eventually pushed peripherally by an erupting tooth. The present case belongs to the former category and the latter type has been identified as “Hybrid variant” [10].

We report the first case of PAOT presenting as a gingival epulis in a Black African female patient with unusual clinical presentation.

## Case presentation

A 25 years old female patient of African ethnicity was referred to the Department of Oral and Maxillofacial Surgery, Ayder Referral hospital (Ethiopia) with the chief complaint of a mandibular gingival mass of two years duration. On general examination, the patient was apparently healthy. The medical history and family history were insignificant. No notable findings were recorded on extra-oral examination.

**Fig. 1:**
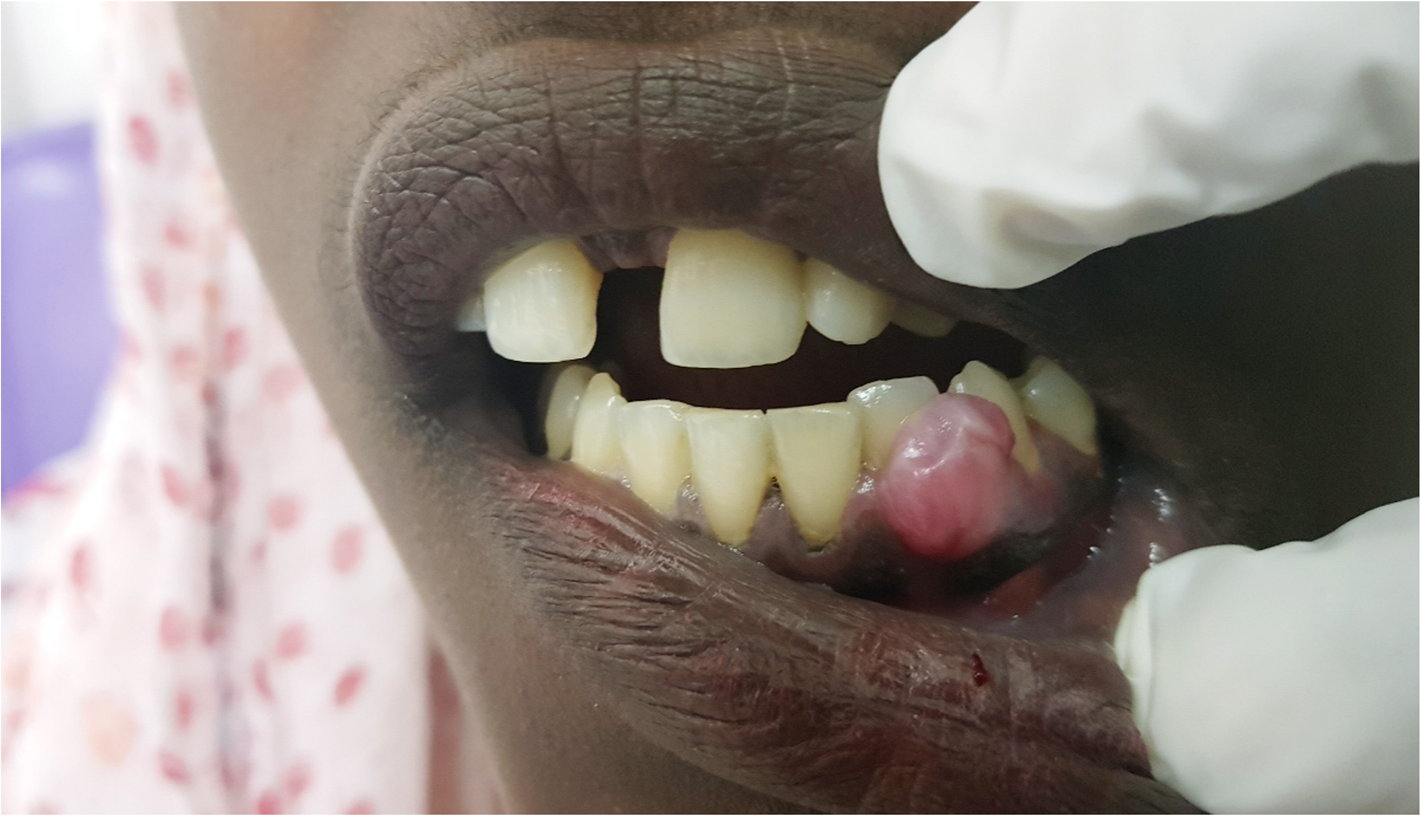
Peripheral adenomatoid odontogenic tumor presenting as a normal colored gingival mass on the anterior mandibular gingiva w.r.t 33 and 34

Intra – oral examination revealed a solitary, well defined, roughly oval shaped gingival mass arising from the attached and free labial gingival margin covering two thirds portion of the crown of teeth 33 and 34 (Fig. 1). It was a slow growing swelling which gradually progressed to its present size of 2 × 2 cm. The overlying mucosa was intact and the color was similar to the adjacent mucosa. Associated signs or symptoms such as pain, bleeding, discharge, numbness or fever were absent; oral hygiene was inadequate. The swelling was non-tender on palpation with firm consistency and smooth surface texture. Intraoral periapical radiograph (IOPA) of right mandibular anterior region was recorded. A minor arc shaped bone loss in relation to teeth 33 and 34 was demonstrated.

Based on the clinico-radiographical findings, clinical diagnosis of pyogenic granulomas was established with differential diagnosis of peripheral ossifying fibroma, peripheral giant cell granuloma and fibrous epulis. The rare differential diagnosis include benign connective tissue tumors and peripheral odontogenic neoplasms.

Following the routine blood examinations, the lesion was removed in toto under local anesthesia. The excised mass was sent for histopathological examination.

Grossly, the specimen was roughly spheroidal in shape measuring 2 × 2 cm approximately and covered by a capsule with soft to firm consistency. Cut section revealed grayish - white appearance with minute hemorrhagic areas.

**Fig. 2:**
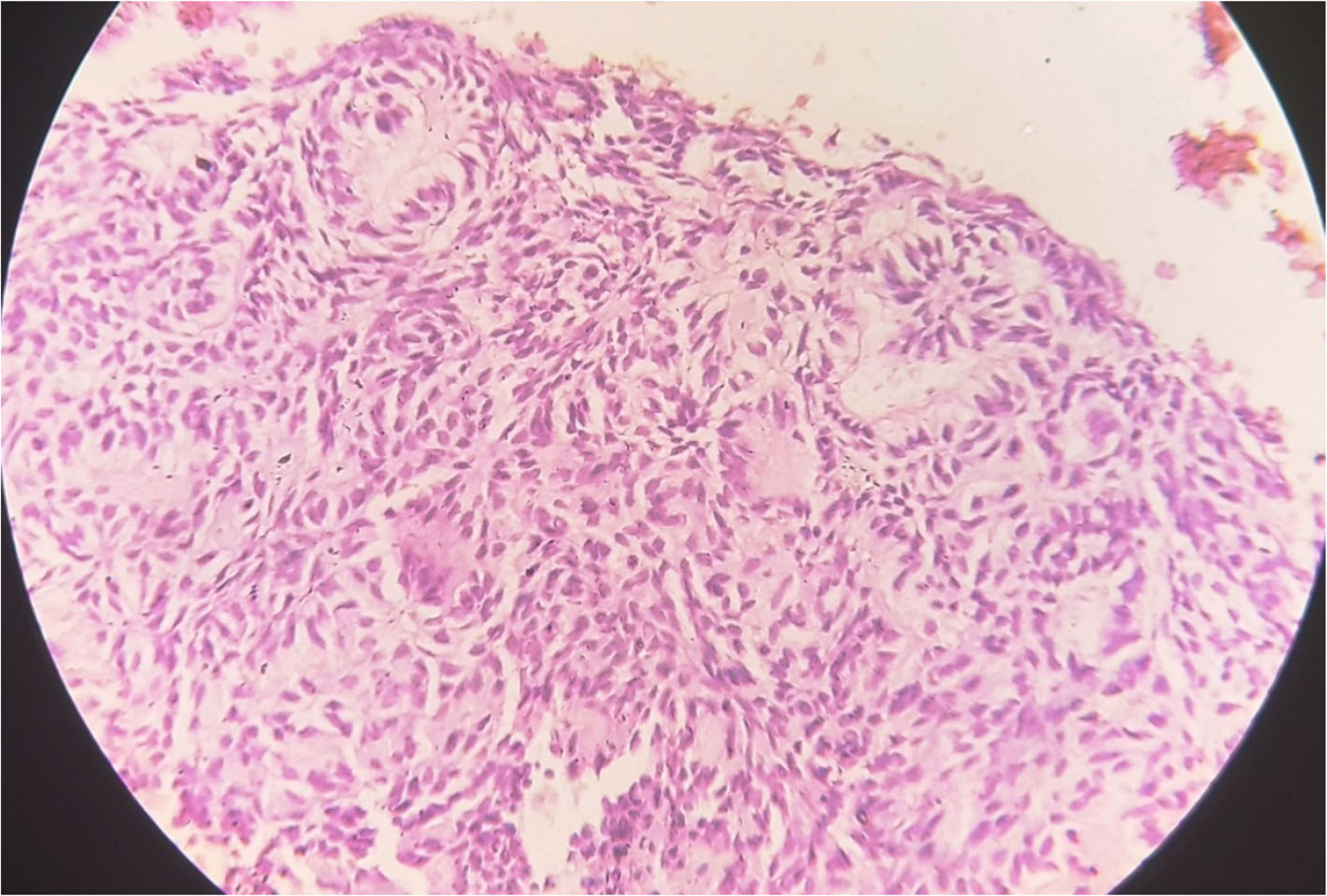
Polyhedral cells arranged in the form of rosettes and whorled pattern; duct like structures are also seen (Hematoxylin and Eosin × 20 magnification)

**Fig. 3:**
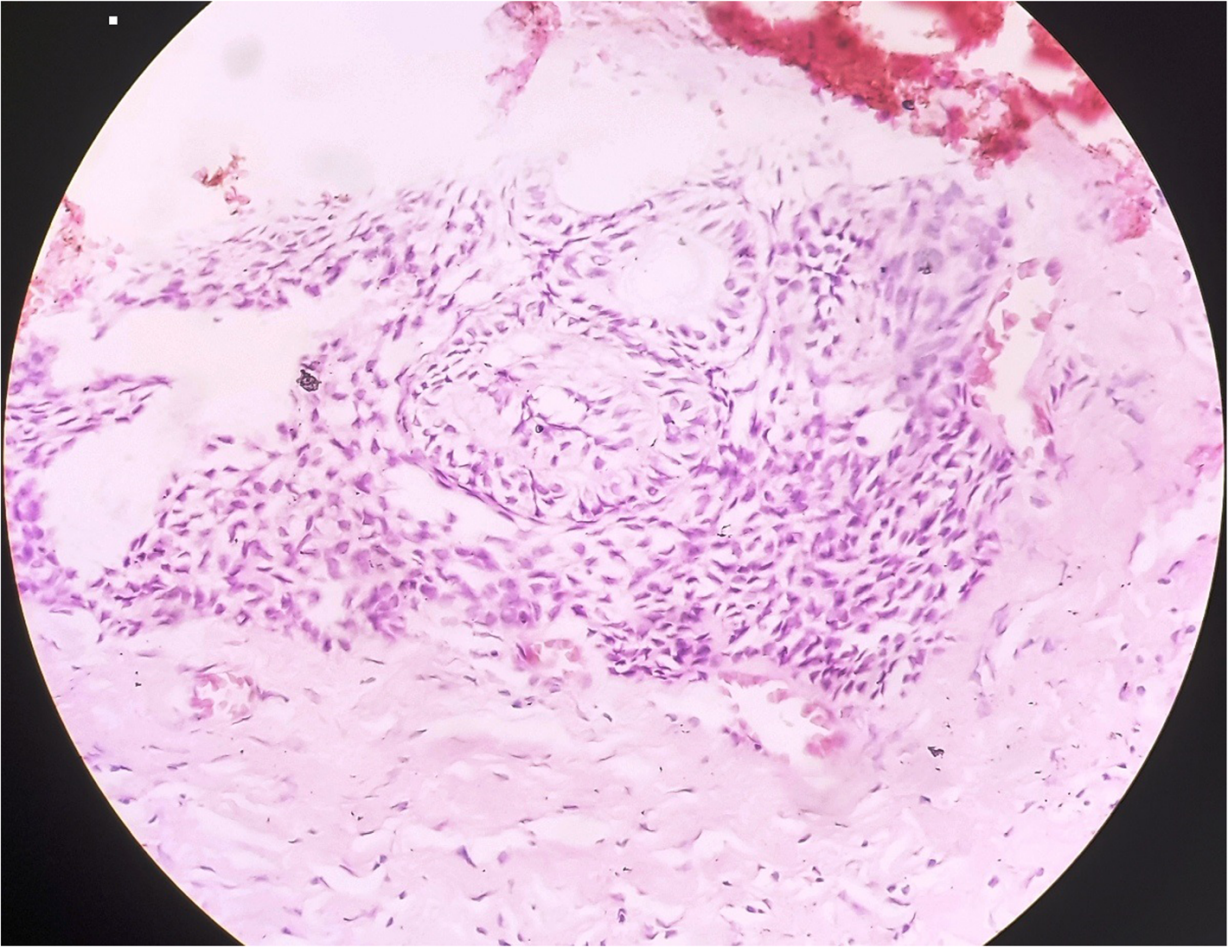
Cuboidal- columnar epithelial cells forming ducts and convoluted double cell rows with surrounding polyhedral cells. An area of cystic degeneration is also seen on the right side (Hematoxylin and Eosin × 20 magnification)

**Fig. 4:**
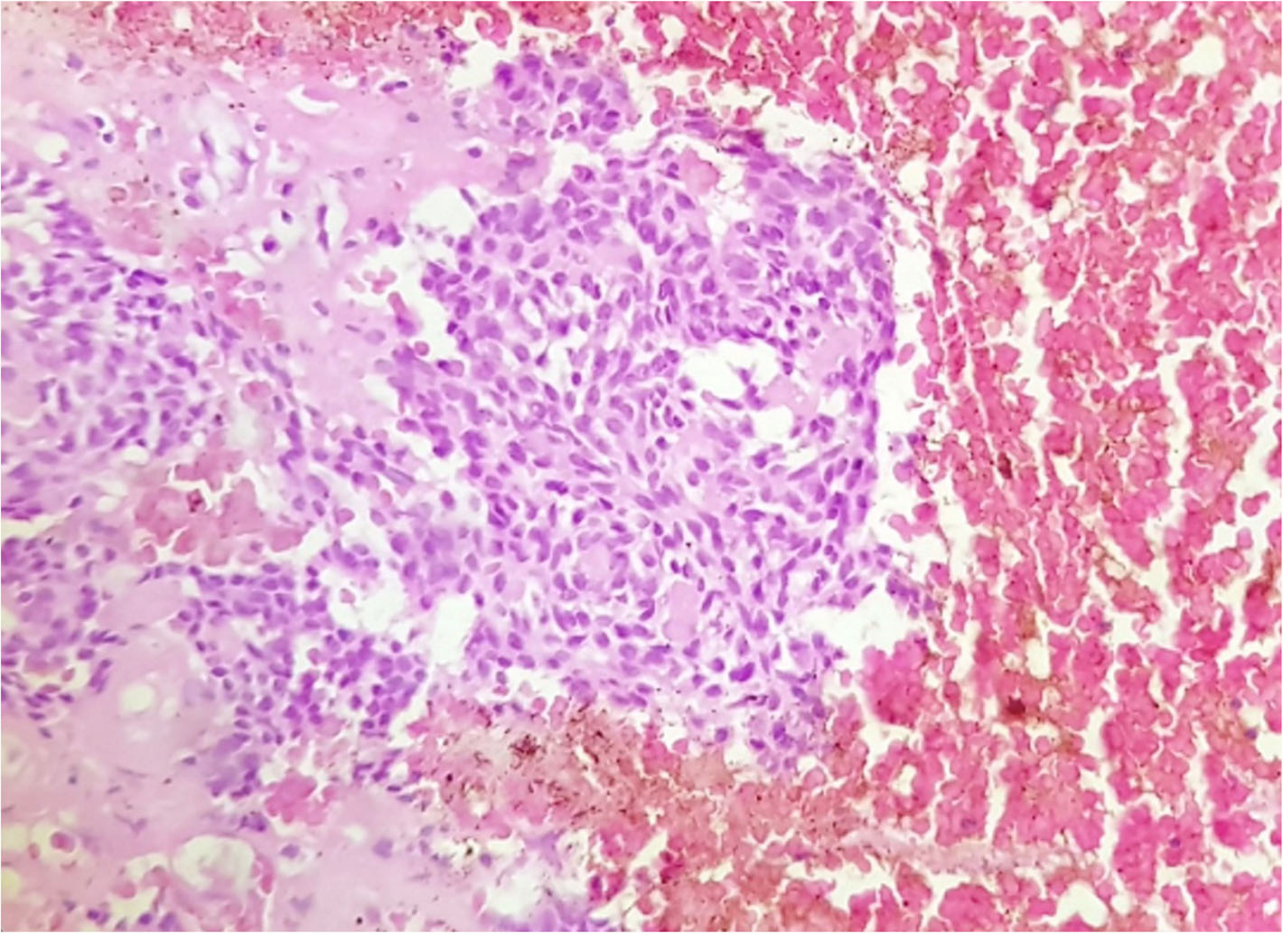
Solid nests of tumor cells containing interspersed eosinophilic droplets in a hemorrhagic background (Hematoxylin and Eosin × 20 magnification)

The haematoxylin and eosin (H&E) stained sections were examined microscopically. The tumor mass was chiefly composed of varied proportions of spindle/polyhedral, cuboidal and columnar cells arranged in multiform patterns with a few areas showing cystic degeneration. Spindle/polyhedral cells were arranged in the whorled pattern; rosette formation was also observed. Cuboidal to tall columnar cells were arranged in the form of microcysts or ducts (Fig. 2). Convoluted rows composed of double layer of columnar cells were also present with an eosinophilic rim of varying thickness between the two layers; these structures were surrounded by proliferation of spindle to polyhedral cells with interspersed eosinophilic material droplets in a hemorrhagic background (Figs. 3 and 4).

On the basis of these classical features, the final diagnosis of peripheral adenomatoid odontogenic tumor was made.

## Discussion and conclusions

Among all the odontogenic tumors, PAOT represent 3.4% of all the tumors. Clinically, it presents as an asymptomatic, slowly but progressively growing gingival colored swelling with a strong predilection for maxillary anterior gingiva. The associated teeth might show some degree of mobility. Analogous to the central varieties, PAOTs also exhibit female predilection with male: female ratio ranging from 1:2 to 1:14 with the majority of cases occurring in younger age group (< 20 years) [1, 9].

We reviewed all the similar case reports available in the literature till date and their details have been summarized in Table 1 [11–26].

**Table 1:** Brief review of PAOT cases published in the literature

Case No.	Author and year	Age (years)	Sex	Site	Size	Disease duration	Radiographic presentation	Treatment	Recurrence	Reference No.
1.	Buchner A et al.,1987	16	F	Maxillary gingiva 1,1	NI^a^	3 years	NI^a^	Conservative surgery	NI^a^	[12]
2.	Buchner A et al.,1987	9	M	Right Maxillary1,2	NI^a^	4 years	NI^a^	Conservative surgery	NI^a^	[12]
3.	Buchner A et al.,1987	11	F	Left Maxillary 1,2	NI^a^	NI^a^	NI^a^	Conservative surgery	NI^a^	[12]
4.	Buchner A et al.,1987	13	F	Mandibular 1,1	NI^a^	4 mo	NI^a^	Conservative surgery	NI^a^	[12]
5	Buchner A et al.,1987	16	F	Right Maxillary 1,2	NI^a^	1mo	NI^a^	Enucleation	NI^a^	[12]
6.	Buchner A et al.,1987	16	F	Left Maxillary 3	NI^a^	2 years	NI^a^	Enucleation	NI^a^	[12]
7.	Unal T et al., 1995	4	F	Right Maxillary 1, gingiva	NI^a^	NI^a^	NI^a^	Enucleation	NI^a^	[14]
8.	Unal T et al., 1995	15	M	Right maxillary 2,3 gingiva	2 × 2 × 2 cm^3^	9 mo	Thickening of laminadura, well-defined radiolucency below 2,3	Curettage	No	[14]
9.	Balwani SR et al., 2007	19	F	Left maxillary 2,3,4 Gingiva	3 × 2.5 × 1.5 cm^3^	4–5 mo	No Bone Involvement	enucleation	NI^a^	[11]
10.	Panjwani S et al., 2010	18	F	Left mandibular 3,4 Gingiva	2 × 2 cm^2^	1 year	Well defined radiolucency	enucleation	Recurrent mass	[16]
11.	Bowers LM et al., 2012	11	M	maxillary 1,1 Gingiva	NI^a^	1 year	Crestal bone loss	enucleation	Recurrent mass	[17]
12.	Kumar R et al., 2012	10	M	Right mandibular 1,2 gingiva	3 × 1.5 cm^2^	3 mo	Well defined radiolucency	Excision	No	[18]
13.	Lavanya N et al.,2013	12	F	Right maxillary 1 gingiva	0.8 × 1.2 × 0.5 cm^3^	NI^a^	Well defined bone defect	Excision	No	[19]
14.	Tavares T et al., 2014	11	F	Left maxillary 1 gingiva	NI^a^.	NI^a^	Diffuse peri-apical radiolucency	enucleation	No	[20]
15.	Jindwani K et al., 2015	21	M	Left maxillary 1,2 gingiva	2 × 2 cm2	1 year	Well defined radiolucency	Enucleation and curettage	N	[21]
16.	Prakash SM et al.,2017	19	F	Right maxillary 2,3 gingiva	2 × 2 cm^2^	6 mo	Arch shaped bone loss	Excision	NI^a^	[22]
17.	Janavi BR et al., 2017	21	F	Left maxillary 1,2 gingiva	1 × 1.5 cm^2^	6 mo	Unilocular Radio Lucency with calcific radio opaque	enucleation	No	[23]
18.	*Melo VDS* et al*, 2014*	14	M	Left maxillary gingiva 2,3	3 cm	4 mo	NI^a^	enucleation	NI^a^	[24]
19.	Mahato et al., 2018	27	M	Right maxillary 3 gingiva	3 × 1.5 × 1.5 cm^3^	1 year	No bone loss	Excision	NI^a^	[25]
20.	Saito A et al., 2018	13	F	Left maxillary incisal gingiva	NI^a^	3 years	insignificant absorption alveolar bone ridge	Excision and enucleation	No	[26]
Case no.	Age (years)	Sex	Site	Size	Disease duration	Symptoms	Radiographic presentation	Treatment	Recurrence	
1.	16	F	Maxillary gingiva 1,1	NI^a^	3 years	NI^a^	NI^a^	Conservative surgery	NI^a^	
2.	9	M	Right Maxillary1,2	NI^a^	4 years	NI^a^	NI^a^	Conservative surgery	NI^a^	
3.	11	F	Left Maxillary 1,2	NI^a^	NI^a^	NI^a^	NI^a^	Conservative surgery	NI^a^	
4.	13	F	Mandibular 1,1	NI^a^	4 mo	NI^a^	NI^a^	Conservative surgery	NI^a^	
5	16	F	Right Maxillary 1,2	NI^a^	1mo	NI^a^	NI^a^	Enucleation	NI^a^	
6.	16	F	Left Maxillary 3	NI^a^	2 years	NI^a^	NI^a^	Enucleation	NI^a^	
7.	4	F	Right Maxillary 1, gingiva	NI^a^	NI^a^	NI^a^	NI^a^	Enucleation	NI^a^	
8.	15	M	Right maxillary 2,3 gingiva	2 × 2 × 2 cm^3^	9 mo	NI^a^	Thickening of laminadura, well-defined radiolucency below 2,3	Curettage	No	
9.	19	F	Left maxillary 2,3,4 Gingiva	3 × 2.5 × 1.5 cm^3^	4–5 mo	Painless swelling	No Bone Involvement	enucleation	NI^a^	
10.	18	F	Left mandibular 3,4 Gingiva	2 × 2 cm^2^	1 year	Painless swelling	Well defined radiolucency	enucleation	Recurrent mass	
11.	11	M	maxillary 1,1 Gingiva	NI^a^	1 year	Painless swelling	Crestal bone loss	enucleation	Recurrent mass	
12.	10	M	Right mandibular 1,2 gingiva	3 × 1.5 cm^2^	3 mo	Painless swelling	Well defined radiolucency	Excision	No	
13.	12	F	Right maxillary 1 gingiva	0.8 × 1.2 × 0.5 cm^3^	NI^a^	Painless swelling	Well defined bone defect	Excision	No	
14.	11	F	Left maxillary 1 gingiva	NI^a^	NI^a^	Painless swelling	Diffuse peri-apical radiolucency	enucleation	No	
15.	21	M	Left maxillary 1,2 gingiva	2 × 2 cm2	1 year	Painless swelling	Well defined radiolucency	Enucleation and curettage	N	
16.	19	F	Right maxillary 2,3 gingiva	2 × 2 cm^2^	6 mo	Painless swelling	Arch shaped bone loss	Excision	NI^a^	
17.	21	F	Left maxillary 1,2 gingiva	1 × 1.5 cm^2^	6 mo	Painless swelling	Unilocular Radio Lucency with calcific radio opaque	enucleation	No	
18.	14	M	Left maxillary gingiva 2,3	3 cm	4 mo	NI^a^	NI^a^	enucleation	NI^a^	
19.	27	M	Right maxillary 3 gingiva	3 × 1.5 × 1.5 cm^3^	1 year	Painless swelling	No bone loss	Excision	NI^a^	
20.	13	F	Left maxillary incisal gingiva	NI^a^	3 years	Painless swelling	insignificant absorption alveolar bone ridge	Excision and enucleation	No	

*M* male, *F* female, ^a^*NI* No Information, *Mo* Month/months

**Fig. 5:**
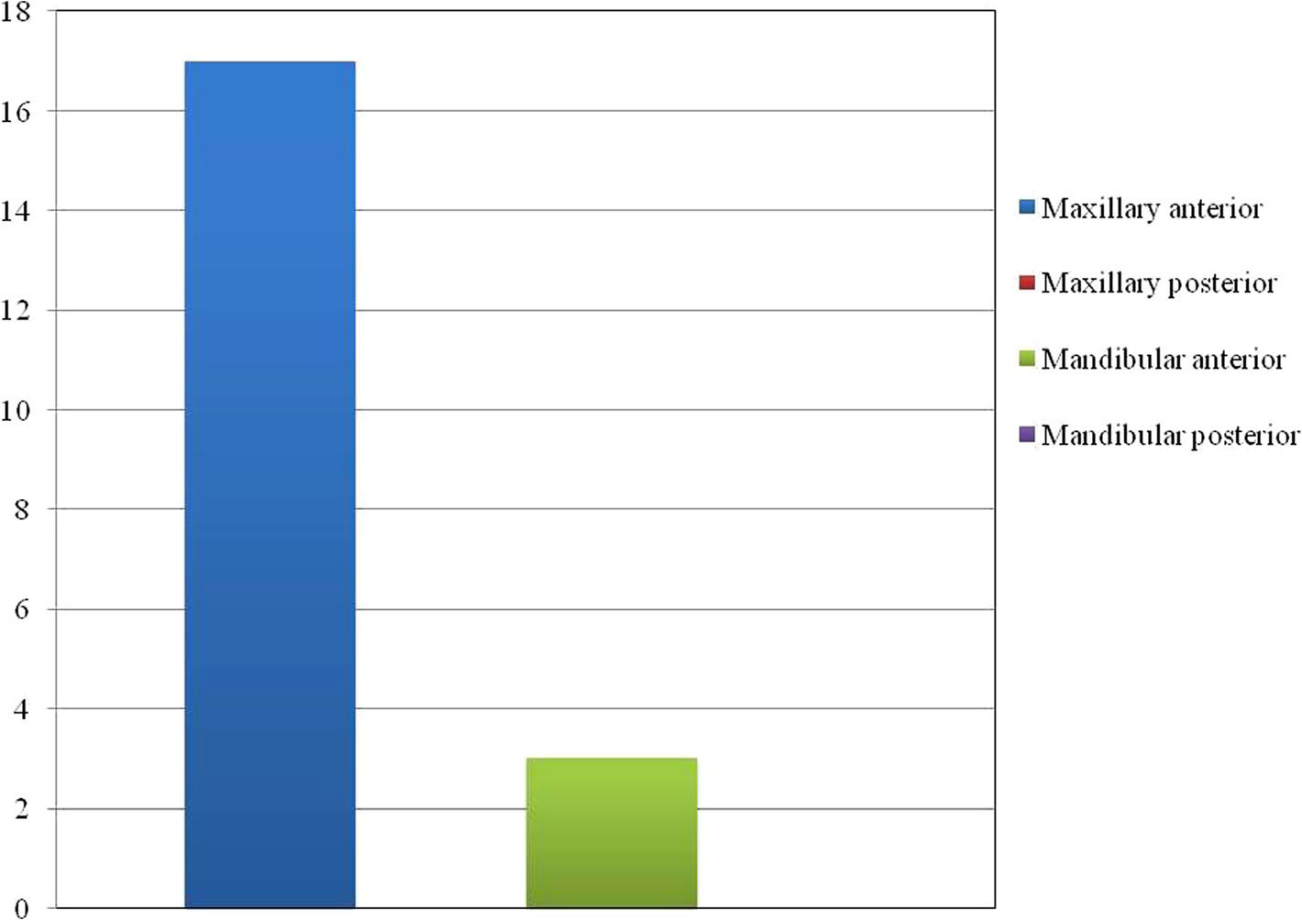
Site distribution of presented PAOT cases

**Fig. 6:**
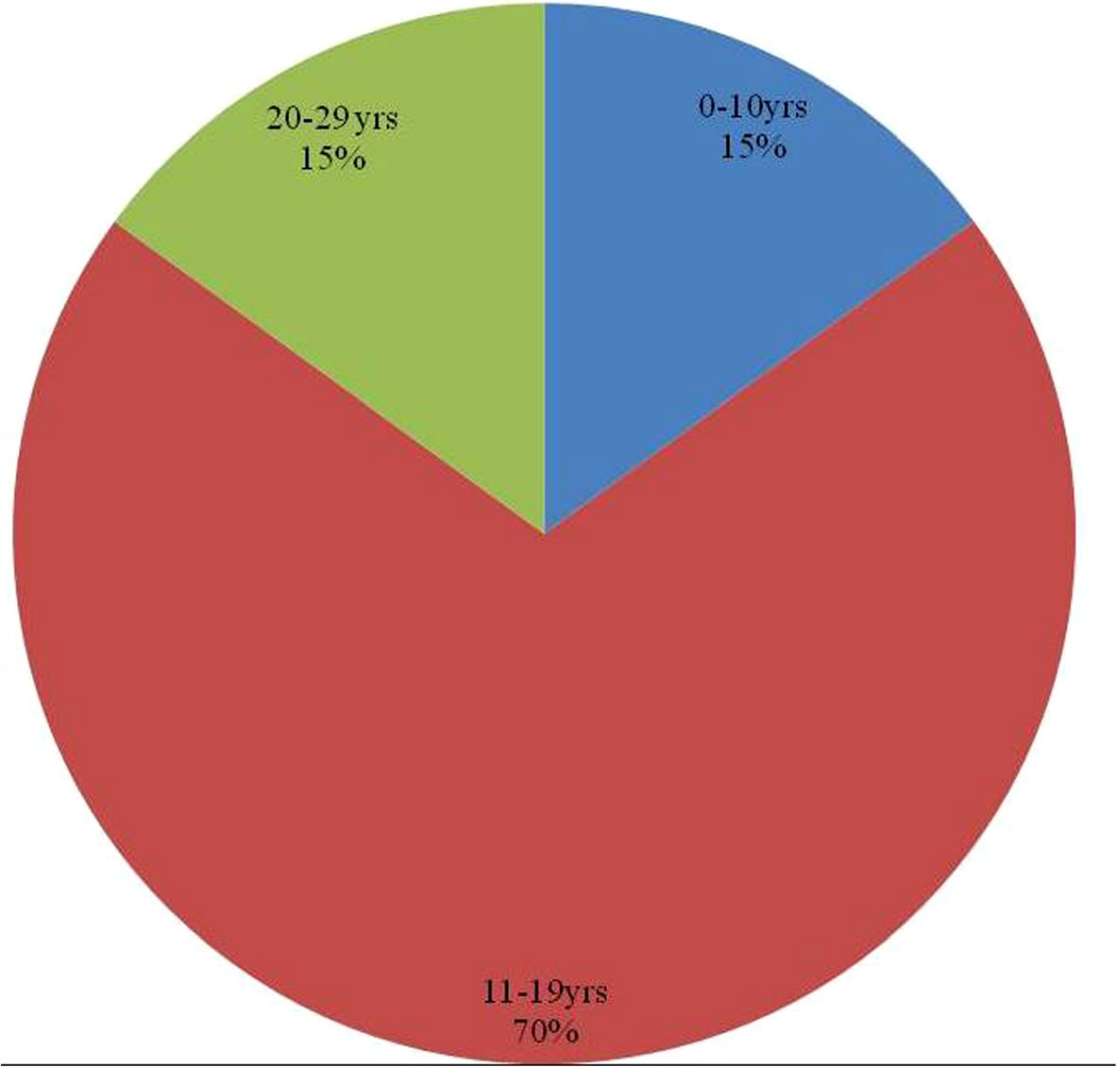
Age distribution of presented PAOT cases

**Fig. 7:**
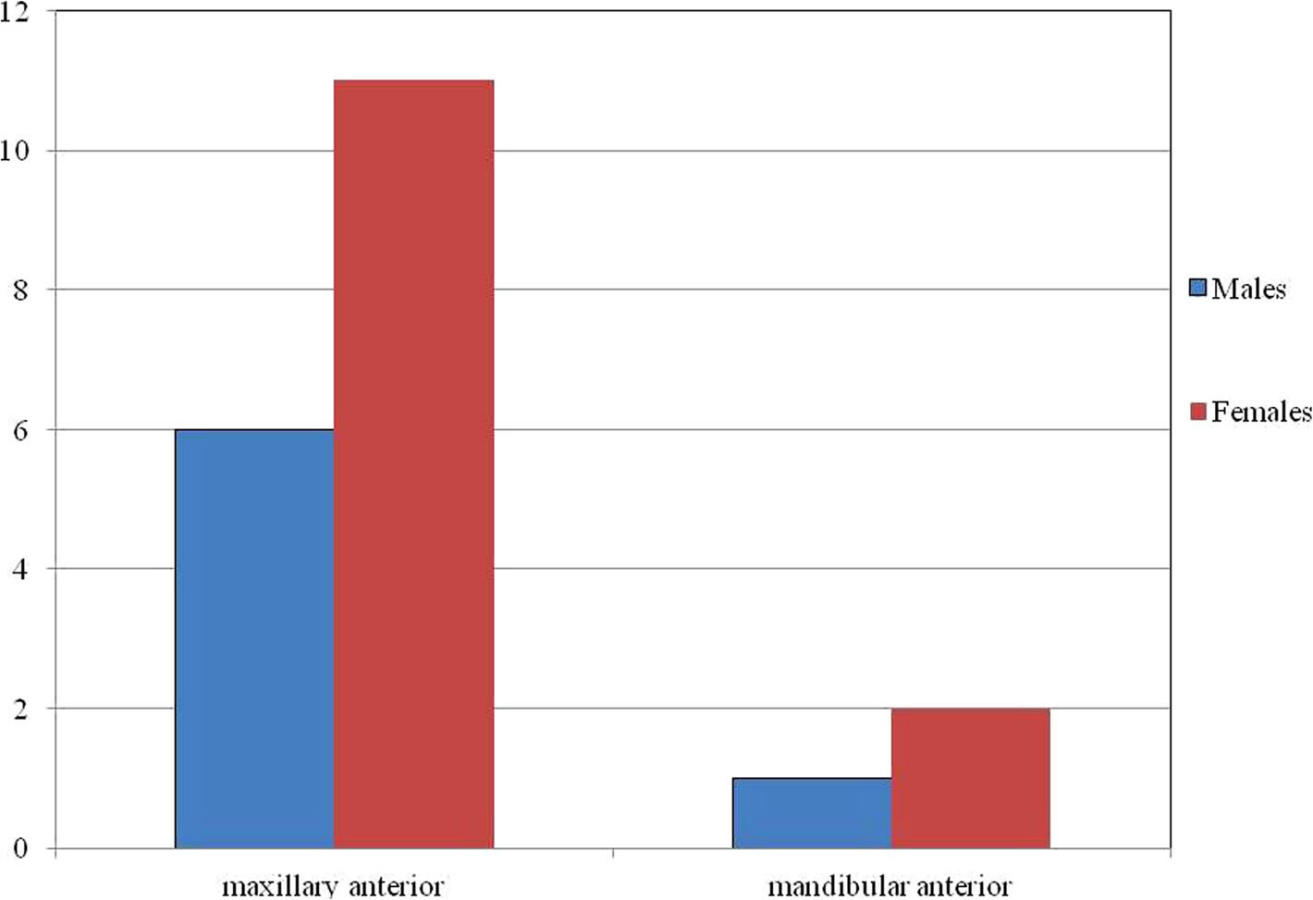
Gender distribution of presented PAOT cases in maxillary and mandibular jaws

Out of the twenty cases reviewed, 17 reveal anterior maxillary presentation indicating the clear predominance for this site followed by 3 cases in anterior mandible; no case has been reported in the posterior segment of the jaws (Fig. 5). The age of occurrence ranges from 4 to 27 years with 70% of the cases seen in second decade (Fig. 6). For both maxilla and mandible, females are almost twice as frequently involved as males; the ratio being male: female:: 1:1.85 (Fig. 7). While most of the cases exhibited the predilection for incisor region only 10% of the cases showed canine-premolar (C-PM) area involvement.

Similar clinical findings were reported by Chrcanovic BR and Gomez RS in 2019 who analyzed the data available on all the variants of AOT [27].

In this report we present the case of PAOT in a 25 years old female patient. Its occurrence in third decade of age and mandibular premolar area involvement makes this case unique. Interestingly, a recent study conducted by Adisa AO et al. in 2016 suggested that there might be slight differences in demography of AOT from region to region and the “Two third tumor” notion generally ascribed to AOT may not be applicable to people of different race or ethnicity uniformly [10]. Also, Sethi et al. reviewed 255 cases of AOT from 2000 to 2014 and observed a striking paradigm shift with respect to prevalence of location [28].

As is evident from the Table 1, most of these tumors present as a painless gingival swelling with the size not exceeding 3 cm and duration varying from one month to four years. Similar findings are observed in the presented case.

Radiographic features varied from negligible to a well defined radiolucency with sclerotic margins or slight erosion of the underlying alveolar bone cortex; minor arc shaped bone loss was seen in the presented case. X ray findings were not available for 8 cases.

Special consideration was given to the CBCT (cone beam computed tomography) findings by Janavi et al. who observed numerous small specks of calcification scattered along the periphery of the lesion in the case of maxillary PAOT. The number, size and degree of calcifications present will influence the radiographic appearance of the lesion [23].

Histologically, the tumor presents with myriad of arrays which are quite characteristic but it is not uncommon to find some Calcifying epithelial odontogenic tumor-like foci. Although present in varying proportions, the tumor is made up of a cellular multinodular proliferation of spindle, cuboidal, and columnar cells in a variety of patterns. Rosette formation, convoluted rows, duct like structures, eosinophilic material, and calcifications are some of its pronounced features. Also, it is invariably covered by a fibrous capsule of variable thickness [4, 8, 9].

Conservative surgical excision with adequate margins is the treatment of choice. In this review, two cases had presented as recurrent tumors [21].

Rightfully called as “master of disguise”, AOT bears a striking resemblance to other more commonly occurring oral lesions. PAOT is often misdiagnosed clinically as one of the simple gingival lesions such as pyogenic granuloma, peripheral giant cell granuloma, peripheral ossifying fibroma and focal mucinosis which often leads to overzealous treatment plans. Diagnosis of PAOT is only uncovered after histopathological investigation [22, 25, 29].

To conclude, we presented the case of black female patient with PAOT having unusual age and site of occurrence; it was initially identified as pyogenic granuloma and treated for the same. No recurrence has been seen since one year follow up of the patient and it is still continued. The authors would like to focus on three important points: (1) Shifting demographic trends of PAOTs with the possible geographical variations (2) POTs should not be overlooked while formulating the differential diagnosis of gingival swellings (3) To reiterate the significance of ‘patient follow- up’ in such cases as the possible biological nature of PAOT is still uncertain, contrary to the innocent behavior exhibited by the other more common gingival lesions resembling PAOT.

The data available on PAOT is clearly inadequate to determinate its distinctive aspects and acknowledge the emerging diversity in the clinical and biological course of this tumor. Hence, authors should be encouraged to report such rare entities.

## Abbreviations

AOT: Adenomatoid odontogenic tumor
CBCT: cone beam computed tomography
H&E: Hematoxylin and eosin
IOPA: Intraoral periapical radiograph
OTs: Odontogenic tumors
PAOT: Peripheral adenomatoid odontogenic tumor
POTs: Peripheral odontogenic tumours
WHO: World Health’s Organization
